# Efficient generation of vesicular stomatitis virus (VSV)-pseudotypes bearing morbilliviral glycoproteins and their use in quantifying virus neutralising antibodies

**DOI:** 10.1016/j.vaccine.2015.12.006

**Published:** 2016-02-03

**Authors:** Nicola Logan, Elizabeth McMonagle, Angharad A. Drew, Emi Takahashi, Michael McDonald, Michael D. Baron, Martin Gilbert, Sarah Cleaveland, Daniel T. Haydon, Margaret J. Hosie, Brian J. Willett

**Affiliations:** a MRC-University of Glasgow Centre for Virus Research, Garscube Estate, Glasgow G61 1QH, United Kingdom; b Royal Veterinary College, University of London, London NW1 0TU, United Kingdom; c Veterinary Diagnostic Services, University of Glasgow, Garscube Estate, Glasgow G61 1QH, United Kingdom; d The Pirbright Institute, Pirbright, Surrey GU24 0NF, United Kingdom; e Wildlife Conservation Society, Bronx, NY, USA; f Boyd Orr Centre for Population and Ecosystem Health, Institute of Biodiversity Animal Health and Comparative Medicine, University of Glasgow, Glasgow G12 8QQ, United Kingdom

**Keywords:** Morbillivirus, Neutralisation, CDV, PPRV, Pseudotype

## Abstract

Morbillivirus neutralising antibodies are traditionally measured using either plaque reduction neutralisation tests (PRNTs) or live virus microneutralisation tests (micro-NTs). While both test formats provide a reliable assessment of the strength and specificity of the humoral response, they are restricted by the limited number of viral strains that can be studied and often present significant biological safety concerns to the operator. In this study, we describe the adaptation of a replication-defective vesicular stomatitis virus (VSVΔG) based pseudotyping system for the measurement of morbillivirus neutralising antibodies. By expressing the haemagglutinin (H) and fusion (F) proteins of canine distemper virus (CDV) on VSVΔG pseudotypes bearing a luciferase marker gene, neutralising antibody titres could be measured rapidly and with high sensitivity. Further, by exchanging the glycoprotein expression construct, responses against distinct viral strains or species may be measured. Using this technique, we demonstrate cross neutralisation between CDV and peste des petits ruminants virus (PPRV). As an example of the value of the technique, we demonstrate that UK dogs vary in the breadth of immunity induced by CDV vaccination; in some dogs the neutralising response is CDV-specific while, in others, the neutralising response extends to the ruminant morbillivirus PPRV. This technique will facilitate a comprehensive comparison of cross-neutralisation to be conducted across the morbilliviruses.

## 1 Introduction

Paramyxoviruses are widespread in nature [1], [2], [3], [4], [5] and species such as bats and rodents harbour many more as yet uncharacterised morbilliviruses with potential for zoonotic spread [6]. While molecular analyses may detect the presence of viral nucleic acid within an organism, studying the serological response of a host species to such pathogens is critical for determining both the level of morbillivirus exposure in a population and the extent of the immune response elicited by both natural exposure and vaccination. However, serological studies are often hampered by the inability to isolate replication competent primary strains of virus. Further, the propagation of primary strains of some morbilliviruses may present a significant biological hazard to either humans or livestock animals, requiring access to high level bio-containment facilities to prevent the inadvertent release of the pathogen into the community. Many of the existing experimental systems for measuring neutralising antibodies to morbilliviruses utilise cell culture adapted strains of virus. During the adaptation process, the biological properties of the virus may alter dramatically to suit the new *ex vivo* environment. For example, the cell-culture adapted strains of measles virus (MeV) such as Edmonston and Hallé attach to target cells by binding to the complement regulatory protein CD46 [7], [8]. In contrast, primary strains of virus require an interaction with signaling lymphocytic activation molecule (SLAM-F1, CD150) [9], [10], [11], [12], a molecule found subsequently to be the primary receptor for all morbilliviruses on lymphoid cells (reviewed in [13]), or nectin-4 (PVRL-4), the cellular receptor for morbilliviruses on epithelial cells [14], [15], [16], [17]. As the receptor binding domain of the morbilliviral haemagglutinin is a target for neutralising antibodies [18], alterations in the receptor binding domain that confer SLAM-independent infection upon cell culture-adapted strains of virus (*e.g.* vaccine strains) may alter the antigenicity of the viral haemagglutinin and thus modulate the sensitivity of the virus to neutralising antibodies. Indeed, while phylogenetically all morbilliviruses are closely related, with each viral species forming a single serotype [19], [20], genotype-specific neutralising antibodies against MeV have been revealed by pre-absorbing sera from naturally infected individuals with cells expressing the haemagglutinin of a vaccine strain of virus [21].

In order to circumvent the use of cell culture-adapted morbilliviruses in live virus neutralisation assays, systems have been developed to facilitate the isolation and culture of primary strains of virus. By engineering Vero cells to stably express the SLAM molecule from the natural host species of the virus [22], [23], the cells provide a substrate with which neutralising antibodies against primary strains of virus may be quantified in live virus-based assays [24]. Unfortunately, the isolation of primary strains of morbilliviruses generally requires access to fresh tissue samples collected *post mortem*, and even then not all primary isolates of virus grow in SLAM-expressing cells [22]. An alternative approach to the measurement of neutralising antibodies is to generate viral pseudotypes bearing both the haemagglutinin (H) and fusion (F) proteins of the virus [9], [22]. While the H protein is the primary target for MeV neutralising antibodies, antibodies recognising the F protein also contribute to a lesser extent to neutralisation [25]. Thus viral pseudotypes bearing both H and F should recapitulate the neutralising response *in toto*. MeV H and F have been expressed successfully on retroviral pseudotypes [26], while canine distemper virus (CDV) H and F pseudotypes have been generated by using a replication-defective vesicular stomatitis virus (VSV) [27]. These “VSVΔG” pseudotypes express the CDV H and F on their surface [22], and thus retain the receptor binding specificity of the primary isolate. Accordingly, they should recreate the sensitivity to neutralising antibody of the parent CDV strain. As the viral particles are derived from VSV, neutralisation assays should not be constrained by the ability of the primary morbillivirus to grow in the target cells. In addition, as the morbilliviral H and F glycoproteins expressed on the VSV pseudotypes are provided *in trans* from transfected plasmids, the amino acid sequences of the H and F are not subject to the antigenic drift that affects stocks of live virus following repeated *in vitro* passage. In effect, viral pseudotypes “freeze the virus in time”, ensuring that the neutralising response is always measured against virions with identical haemagglutinins, facilitating comparisons between animals, viruses and sampling dates. Moreover, neutralising determinants may be mapped on the viral glycoproteins by site-directed mutagenesis.

Globally, CDV is the second most common cause of death due to infectious disease in domestic dogs. However, CDV is not solely a pathogen of dogs, it is a widespread pathogen of carnivores [28], infecting also ferrets, martens, lions, hyenas, tigers, seals and primates [1], [2], [3], [4], [5], [29], [30], [31], [32], [33]. The catastrophic effects of CDV infection on endangered lion, tiger and giant panda populations [30], [34], [35] have heightened awareness of CDV as an ever-present threat to increasingly fragile ecosystems. In contrast, the prevalence of PPRV appears to be increasing globally, with recent outbreaks in Tibet and China (2007) and across North Africa from Morocco to Tunisia (2008–2011) [36], [37]. PPRV causes a devastating disease in small ruminants, threatening both food security and the livelihoods of smallholders. Whether the increase in PPRV is linked to the global eradication of rinderpest, an example of a virus invading a vacated niche [38], remains to be established, however the threat posed by PPRV to the global livestock industry is now significant. Recent outbreaks in Turkey have increased concern that PPRV may spread westward across Europe [39]. If the global threat from viruses such as CDV and PPRV is to be contained, improved rapid, sensitive and specific diagnostic assays are required. Such assays will inform future vaccination programmes, identify potential host populations that might be targeted for surveillance and provide an early warning of impending outbreaks of infection.

The measurement of neutralising antibodies to CDV is routinely performed using live virus microneutralisation tests, a test format that is slow and relies on the visual recording of a cytopathic effect. In contrast, the majority of PPRV antibody tests use enzyme-linked immunosorbent assays (ELISAs), primarily because PPRV microneutralisation tests are labour-intensive and require access to high-level biocontainment facilities. Both CDV and PPRV neutralisation assays are limited to a small number of cell culture-adapted strains of virus. As such, there is currently no means to assess accurately whether there are qualitative differences in the specificity of the neutralising antibodies present in either convalescent sera or sera from vaccinates. Our aim was to develop a flexible system with which we could measure neutralising responses against diverse strains of virus rapidly and with a high sensitivity. Here, we describe the production of VSVΔG pseudotypes bearing CDV H and F glycoproteins and their use in assays for neutralising antibodies. By coupling VSVΔG(CDV) with SLAM expressing target cells, we demonstrate that morbillivirus pseudotypes may be used for the sensitive, rapid and reliable measurement of virus neutralising antibodies.

## 2 Materials and methods

### 2.1 Cell lines and viruses

HEK293 [40] and HEK293T cells (henceforth referred to as 293 and 293T) were maintained in Dulbecco's Modified Eagle Medium (DMEM) supplemented with 10% foetal bovine serum, 100IU/ml penicillin, 100 μg/ml streptomycin, 2 mM glutamine and 0.11 mg/ml sodium pyruvate (complete medium). Medium for 293T cells was supplemented with 400 μg/ml G418 (Geneticin^®^, Life Technologies Ltd.). Vero (African green monkey) cells [41] were maintained in complete medium while Vero-dogSLAM cells were maintained in complete medium supplemented with 400 μg/ml G418. All media and supplements were obtained from Life Technologies Ltd., Paisley, UK. The Onderstepoort strain of CDV was obtained from a proprietary DHPPi vaccine (Nobivac, Merck Animal Health), the A94-11-14, A91-11-15 [42] and A92-27-4 [43] strains were obtained from Ed Dubovi, Cornell University. A94-11-14 and A91-11-15 were propagated in mitogen-stimulated canine peripheral blood mononuclear cells while the Onderstepoort and A92-27-14 strains were maintained in Vero cells. The Snyder Hill strain of CDV [44] and Vero-dogSLAM cells [45] were obtained from Louise Cosby, Queens University Belfast; the Snyder Hill strain was propagated in Vero-dogSLAM cells.

### 2.2 Serum samples

Sera submitted previously to the Veterinary Diagnostic Services laboratory at the University of Glasgow for post-vaccination CDV antibody titre testing were derived from domestic dogs in the United Kingdom. Additional samples were obtained from dogs and lions in Tanzania and diverse mesocarnivore species in Russia. Ruminant sera were collected from ruminants in Tanzania, and experimentally infected ruminants.

### 2.3 Eukaryotic expression vectors and recombinant viruses

The recombinant vesicular stomatitis virus (VSV) in which the glycoprotein (G) gene has been deleted (VSVΔG) and replaced with firefly luciferase (*luc*) has been described [27], [46] and was kindly provided by Michael Whitt, Memphis, TN, USA. An initial stock of VSVΔG*luc* bearing VSVG was used to infect 293T cells transfected with the VSV-G expression vector pMDG [47]. VSVΔG*luc* (VSVG) pseudotypes were recovered, titrated on 293T cells and used to prepare a working stock of VSVΔG*luc* (VSV-G) pseudotypes. To prepare CDV H and F expression constructs, viral RNA was prepared from CDV pelleted by centrifugation (QIAamp Viral RNA Mini kit, Qiagen), used to prepare first strand cDNA (Transcriptor First Strand cDNA Synthesis Kit, Roche), and then used as template in PCR reactions with the following primers: CDV H Sal: 5′-GTCGAC-ACC-ATGCTCCCCTACCAAGACAAGGT-3′, CDV H Not: 5′-GGGCGGCCGC-TTAACGGTTACATGAGAATCTTA-3′: CDV F Sal: 5′-GGGTCGAC-ACC-ATGCACAGGGGAATCCCCAAAAG-3′ and CDV F D633 Not: 5′-GGGCGGCCGC-TTGCTAGCGTCTTTTACAACAGTAAATCAGCA-3′ (Expand High Fidelity PCR system, Roche). Peste des petits ruminants (PPRV) H and F cDNAs were amplified from the Nigeria 75/1 encoding plasmid pCI-PPRV-delL [48] using the primers PPRV-H-NotWtF: 5′-CCGGCGGCCGCACCATGTCCGCACAAAG-3′, PPRV-H-BamH1R: 5′-GGGGGATCCTCAGACTGGATTACATGTT-3′, PPRV-F-Wt-NotF: 5′-GGGGCGGCCGCACCATGCATGCGCCGA-3′, PPRV-F-BamH1-WtR: 5′-GGGGGATCCGCCTACAGTGATCTCACGT-3′, PPRV-F-BamH1-DΔ633R: 5′-GGGGGATCCTGGTTATCTCCCCTTACAG-3′. Amplifications were performed with the following thermocycling conditions: denaturation at 94 °C for 5 min, followed by 35 cycles of 94 °C for 30 s, annealing at 50 °C for 60 s and extension at 72 °C for 120 s, with a final extension at 72 °C for 10 min. Products were digested with the enzymes *Sal*I and *Not*I and cloned into the eukaryotic expression vector VR1012 (Vical Inc.). Canine SLAM-F1 (dogSLAM) was amplified from total RNA prepared from mitogen-stimulated canine peripheral blood mononuclear cells using PCR (Q5^®^ High-Fidelity DNA Polymerase, New England Biolabs) and the primers dogSLAM Bgl: 5′-GCTCAGATCTGAGAGCTTGATGAATTGCCCAG-3′ and dogSLAM Sal: 5′-GCTCGTCGACGCTCTCTGGGAACGTCAC-3′. Amplifications were performed with the following thermocycling conditions: denaturation at 98 °C for 30 s, followed by 30 cycles of 98 °C for 10 s, annealing at 65 °C for 30 s and extension at 72 °C for 60 s, with a final extension at 72 °C for 2 min. The amplified cDNA was cloned into the pDisplay eukaryotic expression vector (Life Technologies, Paisley, UK) using *Bgl*II and *Sal*I. The nucleic acid sequences of all amplified cDNAs were determined externally by Sanger dideoxy chain termination sequencing (*LIGHTrun* Sequencing Service, GATC Biotech AG, Cologne, Germany). All oligonucleotide primers were obtained from Integrated DNA Technologies, Leuven, Belgium.

To prepare VSVΔG*luc* (CDV) and VSVΔG*luc* (PPRV) pseudotypes, 293T cells were transfected with the H and F expression vectors from CDV and PPRV respectively, followed by super-infection with VSVΔG*luc* (VSVG) as described [27], [46]. Supernatants were harvested 48 h post-infection, aliquoted and frozen at -80 °C. The titre of each viral pseudotype stock was estimated by preparing serial dilutions in triplicate and plating onto 293dogSLAM cells followed by incubation for 48–72 h at 37 °C, at which time luciferase substrate was added (Steadylite plus™, Perkin Elmer) and the signal analysed on a Microbeta 1450 Jet luminometer (Perkin Elmer). The viral titre (50% tissue culture infectious dose (TCID) was calculated using the Spearman–Kärber formula [49].

### 2.4 Canine SLAM expressing 293 cells

To prepare target cells for the VSV-ΔG(CDV) pseudotypes, 293 cells were transfected with pDisplay-dogSLAM using linear polyethylenimine, MW 25,000 (Polysciences Inc., Park Scientific, Northampton, UK) and selected in complete medium supplemented with 800 μg/ml G418. The stably transfected 293-dogSLAM cells were expanded and the surface expression of SLAM confirmed by flow cytometry using rabbit polyclonal anti-HA tag (Sigma, Poole, UK) followed by phycoerythrin (PE)-conjugated goat anti-rabbit IgG (Sigma) on a BD Accuri flow cytometer (BD, Oxford, UK). SLAM-F1-expressing cells were compared with cells stably-transfected with the empty vector. The analysis gate was set such that <1% of vector-only control cells were deemed positive.

### 2.5 Live virus microneutralisation assay

Neutralising antibodies were determined by live virus neutralisation assay [50] on Vero cells using the Bussell variant of the Onderstepoort strain of CDV [51]. Four-fold dilutions of each serum sample (ranging from a dilution of 1:16 to 1:16384) were prepared in quadruplicate in complete medium in a 96-well flat bottom plate. ∼100 TCID_50_ of virus was added to each serum dilution and incubated in the dark at room temperature for 1 h, followed by a further hour at 4 °C. 1 × 10^4^ Vero cells were then added to each well and the plates were incubated at 37 °C for three days prior to microscopic examination for cytopathicity.

### 2.6 Pseudotype-based neutralisation assay

2 × 10^4^ 293-dogSLAM cells were plated into each well of a 96-well white flat-bottomed plate (Culturplate-96, Perkin Elmer, Coventry, UK). Four-fold serum dilutions were prepared in triplicate in complete medium ranging from 1:8 to 1:32768. The diluted serum samples were then added to the 293-dogSLAM cells followed by 2.5 × 10^3^ TCID50 of VSVΔG(CDV) pseudotype. Plates were incubated for 48–72 h at 37 °C, at which time luciferase substrate was added (Steadylite plus™, Perkin Elmer) and the signal analysed on a Microbeta 1450 Jet luminometer (Perkin Elmer). Antibody titres were calculated by interpolating the point at which there was a 90% reduction in luciferase activity (90% neutralisation, inhibitory concentration 90 or IC_90_) [52].

## 3 Results

**Fig. 1:**
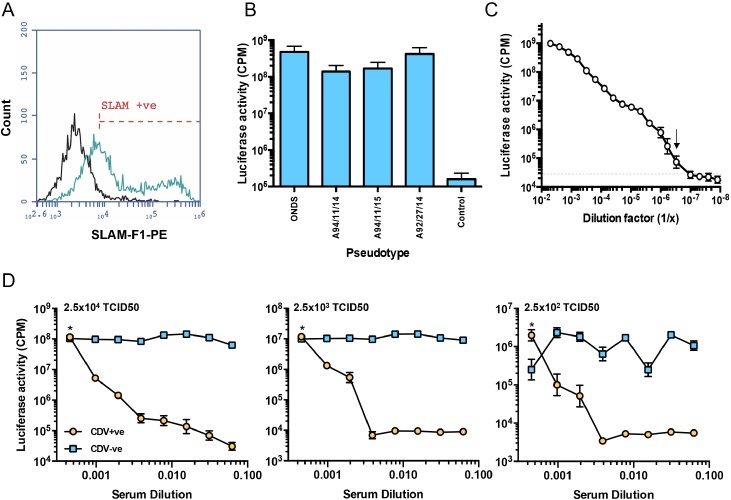
Infection of 293-dogSLAM cells with VSVΔG(CDV) pseudotypes. (A) 293 cells were stably transfected with a canine SLAM expression vector to generate “293-dogSLAM” cells. Expression of the HA-tagged SLAM was confirmed by flow cytometry using rabbit anti-HA tag (blue line) in comparison with vector only control cells (black line). (B) VSVΔG(CDV) pseudotypes bearing the Onderstepoort (ONDS), A94/11/14, A94/11/15 and A92/27/14 H and F proteins, or bearing no H and F proteins (control), were prepared in parallel and plated onto 293-dogSLAM. Luciferase activity was measured 72 h post infection. (C) Titration of VSVΔG(CDV) (Onderstepoort) on 293-dogSLAM. Serial two-fold dilutions of the pseudotypes were prepared in quadruplicate and plated on to 293-dogSLAM. Luciferase activity (CPM) was read at 72 h post-infection, the TCID50 was then calculated using the Spearman–Kärber method. The last luciferase positive dilution representing the end-point of the titration is arrowed. (D) Neutralisation of VSVΔG(CDV) pseudotypes by a CDV-positive serum. CDV-positive and negative sera were incubated with three input amounts of viral pseudotypes, equivalent to 2.5 × 10^2^, 2.5 × 10^3^ and 2.5 × 10^4^ TCID50/well in triplicate and plated onto 293-dogSLAM cells, luciferase activity (CPM) was read at 72 h post-infection. * denotes no serum control, arbitrarily assigned a dilution of 0.0005). Each point represents mean ± SE (*n* = 3, B and D; *n* = 4, C). (For interpretation of the references to colour in this figure legend, the reader is referred to the web version of this article.)

VSVΔG(CDV) pseudotypes bearing a green fluorescent protein (GFP) reporter gene were used previously to define determinants of viral cell tropism [22]. In order to facilitate high throughput screening of sera for neutralising antibodies, VSVΔG(CDV) pseudotypes were prepared carrying a firefly luciferase marker gene (*luc*). As expression of the luciferase reporter gene was very low in Vero-dogSLAM cells, target cells were generated based on 293 cells. Stable transfection and expression of canine SLAM was confirmed by flow cytometry (65.4% SLAM positive, Fig. 1A). Next, the susceptibility of the 293-dogSLAM cells to infection with VSVΔG(CDV) pseudotypes bearing H and F proteins from the Onderstepoort, A94-11-14, A94-11-15 and A92-27-4 strains was investigated (Fig. 1B). Of the four strains tested, the two Vero cell-adapted isolates (Onderstepoort and A92-27-4) gave higher activities consistently, while the two PBMC-grown viruses gave lower mean activities. SLAM-dependent infection of the 293-dogSLAM cells was confirmed by comparison with 293 cells transfected with pDisplay only (Supplementary Fig. 1). Titration of VSVΔG(CDV) Onderstepoort pseudotypes by serial dilution on 293-dogSLAM cells indicated that ∼1 × 10^9^ CPM of luciferase activity equated to ∼1.1 × 10^7^ TCID_50_ (Fig. 1C).

Next, the application of the VSVΔG(CDV) pseudotypes to assays for virus neutralising antibodies was investigated. Three input amounts of VSVΔG(CDV) Onderstepoort strain pseudotypes were compared (Fig. 1D), equivalent to 2.5 × 10^2^, 2.5 × 10^3^ and 2.5 × 10^4^ TCID_50_ per well, our objective being to minimise the volume of viral pseudotypes required per assay while ensuring the accuracy of the determination of antibody titre. Irrespective of the amount of input virus, the measured antibody titre (90% neutralisation) remained consistent, mean titres of 966, 922 and 970 for inputs of 2.5 × 10^2^, 2.5 × 10^3^ and 2.5 × 10^4^ TCID_50_ per well respectively. However, given the variability observed at the lowest input of virus (2.5 × 10^2^ TCID_50_), we elected to use an input of 2.5 × 10^3^ TCID_50_ per well, equivalent to approximately 1 × 10^7^ CPM of luciferase activity.

**Fig. 2:**
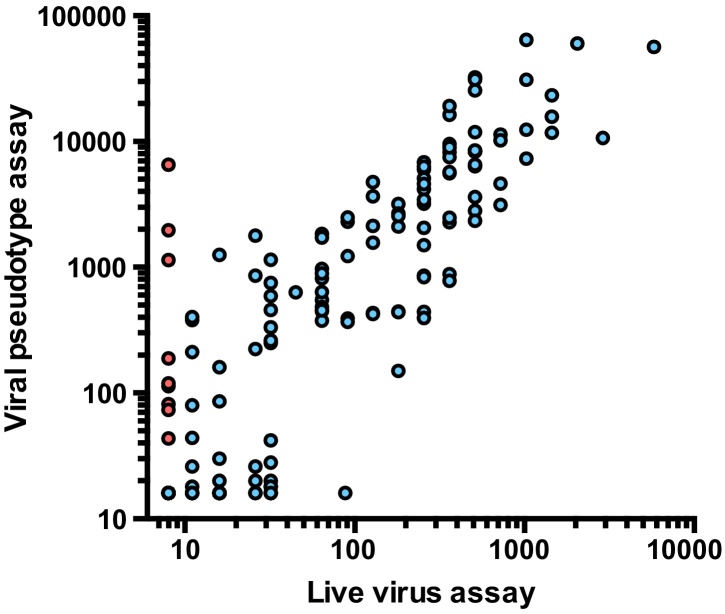
Comparison of neutralising antibody titres calculated using replication competent CDV virus (Bussell strain of Onderstepoort) and VSVΔG (CDV) pseudotypes expressing the Onderstepoort H and F glycoproteins from the Nobivac DHPPi vaccine. 9 of the 44 samples that scored negative by live virus assay (titre <8) were found to have detectable neutralising antibody activity by pseudotype assay (red circles) suggesting that the pseudotype assay facilitated the detection of a number of false negative samples. Spearman *r* coefficient 0.76 (95% confidence interval 0.69–0.81), *p* < 0.0001, *n* = 202. (For interpretation of the references to colour in this figure legend, the reader is referred to the web version of this article.)

CDV neutralising antibody titres in 190 serum samples submitted to the University of Glasgow Companion Animal Diagnostic laboratory for diagnostic testing were compared between a classical “live virus” microneutralisation assay [50] on Vero cells (endpoint titre calculated using Spearman–Kärber method) and VSVΔG(CDV) pseudotypes on Vero-dogSLAM cells (90% reduction in luciferase activity) [52]. The two assays correlated well (Fig. 2), revealing a Spearman *r* coefficient of 0.76 (95% confidence interval 0.69–0.81, *p* < 0.0001), suggesting that the pseudotype assay was a reliable substitute for the live virus-based assay. In general, titres obtained with the pseudotype-based assay were ∼10-fold higher than those obtained with the live virus based assay. Of the 44 samples that scored negative by live virus assay (titre <8), 9 (20%) were found to have detectable neutralising antibody activity by pseudotype assay (red circles, Fig. 2). Given that the neutralising activity in the 9 samples was confirmed to be CDV-specific (no neutralising activity was detected against VSVΔG(VSV-G) pseudotypes), these data suggest that the enhanced sensitivity of the pseudotype assay and capacity for inclusion of unrelated virus controls may facilitate the discrimination of false negatives. At present, live virus microneutralisation tests for CDV antibody lack the capacity for confirmation of the specificity of the test.

34 sera that scored weakly positive by the live virus based assay (titres in the range 11–88, median value 16) were found to have titres of <16 by the pseudotype assay. Given the enhanced sensitivity of the pseudotype-based assay, we would predict that any sample yielding a low titre with the live virus microneutralisation test would give a higher titre with the pseudotype-based test. Accordingly, the finding that the 34 sera reported as weakly positive in the live virus microneutralisation test were negative by the pseudotype test may indicate that these samples represented false positives by the live virus microneutralisation assay. If the 34 sera that scored weakly positive by live virus assay are excluded from the comparison between the tests the correlation between the two assays rises to *r* = 0.91 (95% confidence interval 0.87–0.93, *p* < 0.0001, *n* = 168).

False positives at high serum concentrations are not uncommon with live virus microneutralisation tests and the definition of a reliable cut-off point in the absence of a “gold-standard” diagnostic test is problematic. A significant concern with live virus-based assays is their reliance on visual inspection of a cytopathic effect within the monolayer of cells for endpoint determination. Non-specific toxic effects of the serum sample, especially at high serum concentrations, may alter the morphology of the monolayer, interfering with the interpretation of the assay end-point. The enhanced sensitivity of the pseudotype based assay reduces the susceptibility of the assay to non-specific toxic effects and accordingly may facilitate the detection of virus neutralising antibodies at lower dilutions of serum, circumventing the potential toxic effects of such sera.

An alternative consideration is that the sera scoring weakly positive by live virus neutralisation test might be reacting with an antigen present on live virus that is absent from VSVΔG(CDV) pseudotypes. As the Bussell strain of CDV used in this study was propagated in Vero (African green monkey kidney epithelial) cells while the VSVΔG(CDV) pseudotypes were generated in 293T (human embryonic kidney) cells, it is conceivable that the weak reactivity detected in the live virus assay reflects an activity against a Vero cell-derived antigen that is absent from the 293T-derived VSVΔG(CDV) pseudotypes. Similarly, as the live virus assay is SLAM-independent, it is also possible that the live virus assay detects a very weak response against distinct epitopes on the viral glycoproteins that are critical for SLAM-independent infection but are rendered insignificant in the presence of a high affinity SLAM-interaction as in the VSVΔG(CDV) pseudotype assay.

**Fig. 3:**
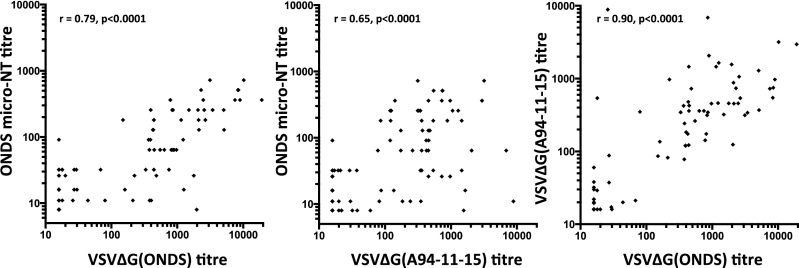
Neutralisation of VSVΔG(CDV) pseudotypes bearing Onderstepoort (ONDS) and A94-11-15 glycoproteins by sera from African dogs only; comparison with classical “live virus” micro-neutralisation test (micro-NT). Each serum was tested in triplicate (VSVΔG(CDV) pseudotype assay) or quadruplicate (micro-NT). Antibody titres against VSVΔG(CDV) pseudotypes were calculated based on 90% reduction of infectivity while micro-NT titres were calculated using the Spearman–Kärber formula. Spearman *r* coefficients were calculated for each plot.

It is widely assumed that antibody responses to CDV form a single serotype, however the majority of assays performed to date have utilised a limited number of Vero cell-adapted strains of virus. If we are to assess accurately the neutralising response against field strains of virus, the assays should be performed using biologically relevant, non-cell culture adapted strains of virus. To investigate the feasibility of this approach we compared the neutralisation sensitivity of VSVΔG(CDV) pseudotypes bearing the H and F proteins of the primary A94-11-15 strain with those bearing the cell culture adapted Onderstepoort strain. A94-11-15 is an isolate from an African dog collected during the 1994 CDV outbreak in the Serengeti [42]. The Onderstepoort and A94-11-15 pseudotypes were screened against a batch of dog sera collected in the Serengeti region between 2007 and 2011. Firstly, a strong positive correlation was observed between the antibody titres estimated using the classical live virus microneutralisation test (ONDS micro-NT) and the pseudotype-based (VSVΔG(ONDS)) test (*r* = 0.79, *p* < 0.0001; Fig. 3). When the analysis was repeated using VSVΔG(A94-11-15) pseudotypes, a significant, but weaker correlation was noted between the titres estimated using the ONDS micro-NT and the VSVΔG(A94-11-15) pseudotypes (*r* = 0.65, *p* < 0.0001; Fig. 3). The ONDS micro-NT measures a reduction in the cytopathic effect observed in Vero cells following infection. In contrast, the VSVΔG pseudotype test measures inhibition of viral entry mediated by canine SLAM. Given that the A94-11-15 strain is a SLAM-dependent field strain while Onderstepoort is a cell culture-adapted, SLAM-independent strain, it is likely that differences would be evident between the two assay systems. When the titres obtained with VSVΔG pseudotypes bearing the ONDS and A9-11-15 glycoproteins were compared, a highly significant correlation was observed (*r* = 0.90, *p* < 0.0001; Fig. 3), perhaps suggesting qualitative and quantitative differences in the neutralising antibodies that are being measured on Vero cells and 293dogSLAM cells.

**Fig. 4:**
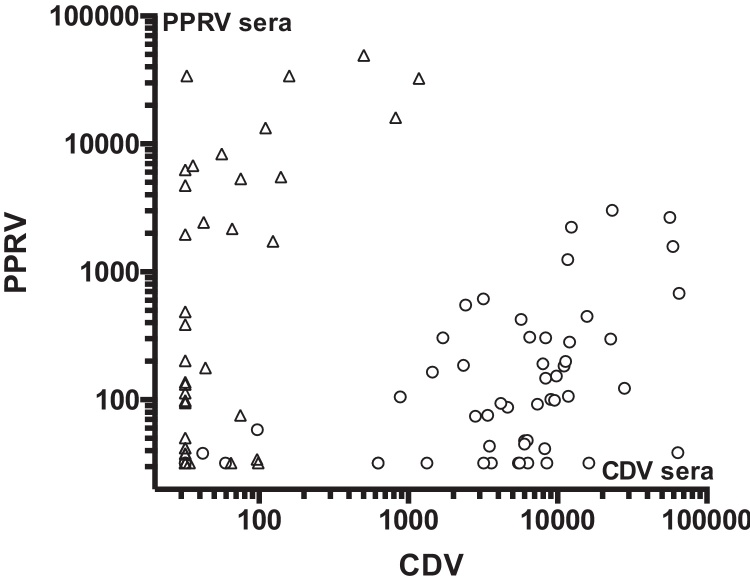
Cross-neutralisation of VSVΔG (CDV) and (PPRV) pseudotypes by sera raised against PPRV and CDV. Sera from ruminants (Δ) exposed or vaccinated against PPRV, or dogs (O) vaccinated against or naturally exposed to CDV were screened for neutralising antibodies against CDV or PPRV. Each point represents the serum titre that reduced luciferase activity by 90% relative to the no serum control as calculated by serial dilution.

In order to define further the viral species-specificity of the pseudotype based test, we compared the neutralisation sensitivity of VSVΔG (CDV) pseudotypes, with pseudotypes prepared using the H and F proteins of the genetically distant morbillivirus, PPRV (peste des petits ruminants). As the degree of antigenic-relatedness varies between morbilliviruses, we would predict that a high titre antibody response against CDV would encompass weaker, cross-neutralising activity against PPRV. Conversely, a high titre anti-PPRV serum would neutralise CDV pseudotypes more weakly. The sensitivities of VSVΔG (CDV) pseudotypes and VSVΔG (PPRV) pseudotypes to neutralisation by sera from animals exposed to, or immunised against, CDV and PPRV was compared. The CDV sera consisted of dog sera from the UK, Tanzania and Russia, and PPRV sera consisted of ruminants from Africa and Asia. The PPRV sera displayed titres of neutralising antibodies that were ∼100-fold higher against VSVΔG (PPRV) than against VSVΔG (CDV) (Fig. 4). Conversely, the CDV sera neutralised VSVΔG (CDV) ∼100-fold more efficiently than VSVΔG (PPRV) (Fig. 4). Using a general linear model (GLM), the log10-transformed neutralising titres of the dog sera against CDV and the ruminant sera against PPRV, were compared with the log10-transformed neutralising titres of the dog sera against PPRV and the ruminant sera against CDV. The results of the GLM showed the difference to be highly significant (*p* < 0.0001). Thus, the specificity of the CDV neutralising response could be confirmed by comparing the responses against CDV with those against PPRV; a higher titre against CDV than PPRV would suggest that the animal was exposed primarily to CDV.

**Fig. 5:**
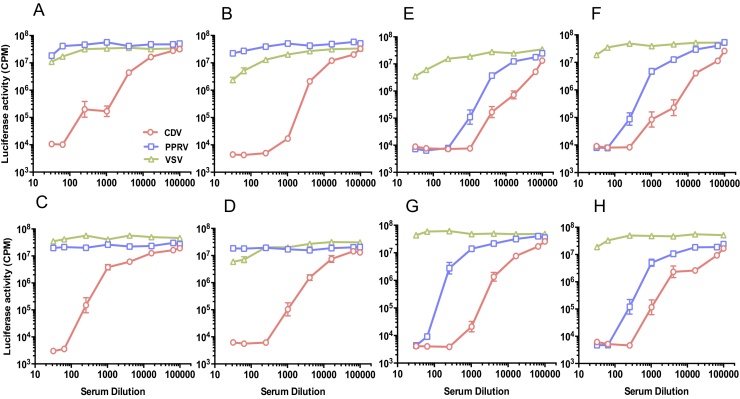
Neutralisation of VSVΔG (CDV), VSVΔG (PPRV) and VSVΔG (VSV) pseudotypes by sera from vaccinated dogs in the UK. While some sera appeared to be CDV-specific (A–D), other sera (E–H) displayed broader neutralising activity, neutralising PPRV pseudotypes effectively, albeit with a lower a titre. Absence of neutralising activity against VSVΔG (VSV) pseudotypes confirmed the specificity of the response. Each point represents mean ± SEM.

Neutralising responses against CDV and PPRV were compared in a collection of post-CDV vaccination sera from dogs (Fig. 5). Given that the sera were derived from dogs in the UK, the animals could not have been exposed to PPRV and any neutralising activity against PPRV would represent a cross-neutralising anti-CDV response. While some sera appeared to be CDV-specific (Fig. 5A–D), other sera displayed strong cross reactivity with PPRV (Fig. 5E–H). These data would suggest the existence of epitopes that are either relatively conserved across species of morbillivirus (as in Fig. 5E–H) or which are specific to a single viral species such as CDV (Fig. 5A–D). As illustrated in Fig. 4, the degree of cross neutralisation varies from animal to animal and may reflect a combination of both the host's ability to mount a humoral response to the virus and the nature of the exposure to the viral antigen. In order to confirm the specificity of the neutralising response further, sera were also tested against VSVΔG (VSV) pseudotypes. Sera that showed neutralising activity against both CDV and PPRV did not neutralise the VSVΔG (VSV) pseudotypes, confirming the specificity of the response. Weak activity against VSVΔG (VSV) observed at high serum concentrations in some samples (*e.g.* Fig. 5B and E) could be confirmed as non-specific inhibitory activity as VSV is not present in the UK. In selecting a stringent 90% reduction in infectivity as the basis for calculation of the antibody titre, such weak, non-specific neutralising activity is eliminated and samples with such activity are scored as negative.

## 4 Discussion

**Fig. 6:**
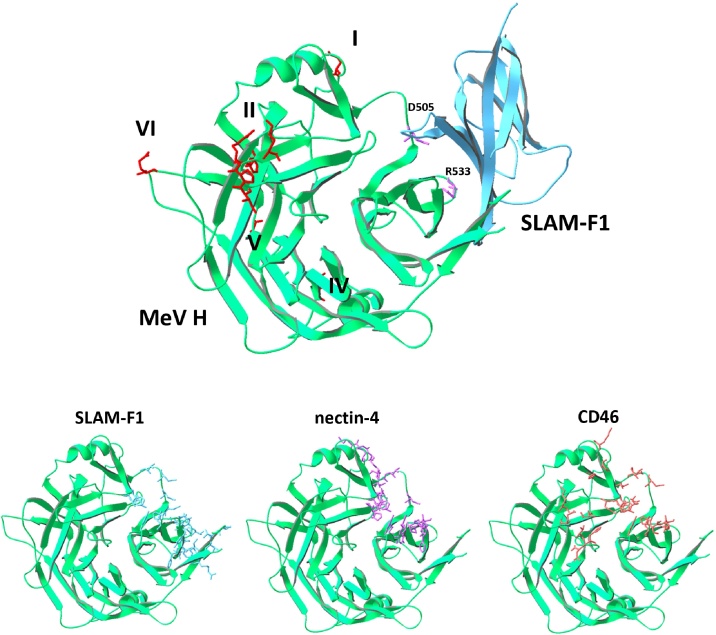
Structure of measles virus (MeV H) haemagglutinin (green), highlighting residues involved in SLAM-F1 binding (cyan), nectin-4 binding (magenta) and CD46 binding (orange). Residues D505 and R533 render measles virus resistant to neutralisation by monoclonal antibody 2F4 and disrupt SLAM-F1 binding [18]. Additional binding sites for measles virus neutralising antibodies have been mapped to regions I, II, IV, V and VI [18], site II overlaps the CD46 binding site only, but not the binding sites for SLAM-F1 or nectin-4.

In this study, we describe the measurement of morbilliviral neutralising antibodies using a viral pseudotype-based assay. The aim of the study was to develop an assay that was rapid, sensitive and which enabled the analysis of neutralising responses against field strains of virus without a requirement for virus adaptation to Vero cell culture. One of the primary concerns with adapting viruses to cell culture is that the cell-culture adapted viruses no longer reflect the neutralisation sensitivity of field strains of the virus. Field strains of virus are generally isolated onto cells expressing the cognate SLAM molecule, while cell culture-adapted strains of virus are able to replicate in SLAM-negative cell lines such as Vero. The nature of the viral receptor utilised by a virus can determine the sensitivity of the virus to neutralising antibodies. Consequently, alterations in receptor usage during adaptation for *in vitro* growth may alter the neutralisation sensitivity of the virus. In regard to the genus *Morbillivirus*, this phenomenon has been observed with measles virus, where neutralising epitopes were mapped to the receptor binding sites on the haemagglutinin of the virus [53] (Fig. 6). For example, the activity of the neutralising monoclonal antibody 2F4, an antibody that targets a conserved neutralising epitope, is disrupted by mutations in residues D505 and R533 of the measles haemagglutinin (Fig. 6) [18]. Mutations in residues D505 or R533 also disrupt the haemagglutinin-SLAM-F1 interaction, suggesting that escape from neutralisation by antibodies such as 2F4 may drive the emergence of SLAM (and nectin-4) independent strains [18]. Accordingly, *in vitro* systems for measuring neutralising antibodies should utilise SLAM-F1, or nectin-4-expressing cell lines if they are to recapitulate fully the expected neutralisation sensitivity of the virus *in vivo*. In this study, we generated canine SLAM-expressing 293 cells as an indicator cell line for the neutralisation assay. The “293dogSLAM” cells expressed the VSVΔG-encoded luciferase gene efficiently. Using these target cells, we generated a luciferase-based assay that was approximately 10-fold more sensitive than the conventional live virus based microneutralisation assay and which yielded data in three days with no requirement for operator interpretation of cytopathic effect by visual inspection. As a glow luciferase-based system, the assay is amenable to high-throughput screening using small sample volumes. Uniquely, the modular nature of the VSVΔG pseudotyping system facilitates the preparation of pseudotypes bearing a range of heterologous glycoproteins from diverse, primary strains of CDV. In proof-of-concept, we prepared pseudotypes bearing H and F glycoproteins from both the Vero cell-adapted Onderstepoort and A92-27-14 strains, and the primary field strains A94-11-14 and A94-11-15. Of the CDV strains tested, the two Vero cell-adapted isolates (Onderstepoort and A92-27-4) yielded higher titres during pseudotype production than the other strains tested, perhaps indicating that adaptation to *in vitro* growth altered the biological properties of these viruses, favouring more efficient pseudotype production. Efficient pseudotyping of morbilliviral glycoproteins on VSV particles requires optimal H and F expression on the plasma membrane of the transfected 293T cells at the site of VSV assembly and budding. Future studies should investigate whether field strains of other morbilliviruses vary in their relative efficiencies of H and F incorporation and whether this impacts upon receptor usage and sensitivity to neutralising antibodies.

Classical live virus microneutralisation assays or PRNT assays rely on the visual inspection of the indicator cells for a cytopathic effect or plaque formation. As such, viral entry events that do not trigger a cytopathic effect or plaque formation will not be quantified and neutralisation of such infectivity will not be recorded. In contrast, the pseudotype based neutralisation assay measures neutralisation of viral entry mediated by the viral receptor SLAM-F1. Accordingly, all successful entry events that lead to gene expression are detected by measuring luciferase activity. Thus, while a live virus assay measures the net result of a complex series of events, from entry, through cell-cell spread and cytopathic effect, a pseudotype-based assay focuses solely on antibodies that prevent viral entry. Given that morbilliviruses have evolved two distinct receptor interactions (SLAM-F1 and nectin-4), it may be possible to distinguish antibodies that prevent cell-free virus infection from those that prevent cell-cell spread and dissemination. Cell-free virus infection would be best modelled using SLAM-F1 expressing target cells whereas the spread of virus across epithelial barriers would be modelled better using nectin-4 expressing target cells. The pseudotype-based assay system will facilitate the dissection of the neutralising response and permit the determination of whether the nature of the viral receptor expressed on the target cell is critical to the outcome of the assay.

In order to assess the specificity of the system we generated pseudotypes bearing the H and F glycoproteins of PPRV (Nigeria 75/1 strain). Sera raised against CDV, either through vaccination or natural exposure, neutralised CDV ∼100-fold more efficiently than PPRV. In contrast, sera raised against PPRV neutralised PPRV pseudotypes ∼100-fold more efficiently than CDV pseudotypes. These observations reveal two things about the assay system. Firstly, the specificity of the CDV response can be confirmed by comparing against a distantly related viral species. The specificity of a low titre response against CDV can be confirmed by analysing in parallel, the response against PPRV. Secondly, the assay system provides a simple means of measuring cross-neutralisation between distinct strains and species of virus. Indeed, while some UK dog sera appeared to display good neutralisation of PPRV, a pathogen absent from the UK, others were largely CDV-specific. These data suggest that the humoral responses of UK dogs to vaccination against CDV differ in their epitope specificities; some dogs recognising determinants that are broadly conserved between CDV and PPRV while other dogs mount a largely CDV-specific response. Recent studies have noted that a proportion of human sera contain weak cross-neutralising antibodies against CDV [54], suggesting that a potent anti-measles response may induce a low level cross-neutralising response to CDV. Our data would suggest that the potency of the heterologous neutralising response is not solely dependent on the homologous antibody titre; rather there are qualitative differences in the breadth of the neutralising response to infection between hosts. Whether such differences reflect the variable nature of the host immune response to vaccination, the antigenicity of the vaccine used to elicit the response, or post-vaccination exposure to field strains of CDV remains to be established. The significance of these observations to the breadth of immunity conferred by vaccination merits further investigation, both from the perspective of CDV vaccination where a broader response may protect against diverse field strains of the virus, and from the perspective of protecting against the threat of zoonotic transmission of morbilliviruses between species.

Several reports have described the cross-species transmission of morbilliviruses, including CDV, PDV and PPRV [1], [2], [3], [4], [5], [29], [30], [31], [32], [33], [34], [55], [56]. With the global eradication of rinderpest, an ecological niche may have been created for related morbilliviruses such as PPRV [57]. Similarly, progress towards the eradication of measles may leave humans exposed to an emerging threat from amphotropic viruses such as CDV [58]. Preventing such zoonotic transmissions presents a major challenge to both human and veterinary medicine. For example, could measles vaccines protect humans from infection with CDV? Recent data would suggest that measles vaccination does provide partial protection to macaques from infection with CDV [24]. If vaccination against one morbillivirus species induced cross-protection against related morbilliviral species, the risks of zoonoses would diminish markedly. Thus, access to assay systems that facilitate the accurate, sensitive and reproducible quantification of cross-neutralising antibodies will play a key role in selecting vaccine compositions and regimen that are most likely to prevent novel morbilliviral epizootics.

## References

[1] Sakai K., Yoshikawa T., Seki F., Fukushi S., Tahara M., Nagata N. (2013). Canine distemper virus associated with a lethal outbreak in monkeys can readily adapt to use human receptors. J Virol.

[2] Sakai K., Nagata N., Ami Y., Seki F., Suzaki Y., Iwata-Yoshikawa N. (2013). Lethal canine distemper virus outbreak in cynomolgus monkeys in Japan in 2008. J Virol.

[3] Qiu W., Zheng Y., Zhang S., Fan Q., Liu H., Zhang F. (2011). Canine distemper outbreak in rhesus monkeys, China. Emerg Infect Dis.

[4] Yoshikawa Y., Ochikubo F., Matsubara Y., Tsuruoka H., Ishii M., Shirota K. (1989). Natural infection with canine distemper virus in a Japanese monkey (*Macaca fuscata*). Vet Microbiol.

[5] Sun Z., Li A., Ye H., Shi Y., Hu Z., Zeng L. (2010). Natural infection with canine distemper virus in hand-feeding Rhesus monkeys in China. Vet Microbiol.

[6] Drexler J.F., Corman V.M., Muller M.A., Maganga G.D., Vallo P., Binger T. (2012). Bats host major mammalian paramyxoviruses. Nat Commun.

[7] Dorig R.E., Marcil A., Chopra A., Richardson C.D. (1993). The human CD46 molecule is a receptor for measles virus (Edmonston strain). Cell.

[8] Naniche D., Varior-Krishnan G., Cervoni F., Wild T.F., Rossi B., Rabourdin-Combe C. (1993). Human membrane cofactor protein (CD46) acts as a cellular receptor for measles virus. J Virol.

[9] Tatsuo H., Ono N., Yanagi Y. (2001). Morbilliviruses use signaling lymphocyte activation molecules (CD150) as cellular receptors. J Virol.

[10] Tatsuo H., Yanagi Y. (2002). The morbillivirus receptor SLAM (CD150). Microbiol Immunol.

[11] Erlenhoefer C., Wurzer W.J., Loffler S., Schneider-Schaulies S., ter Meulen V., Schneider-Schaulies J. (2001). CD150 (SLAM) is a receptor for measles virus but is not involved in viral contact-mediated proliferation inhibition. J Virol.

[12] Hsu E.C., Iorio C., Sarangi F., Khine A.A., Richardson C.D. (2001). CDw150(SLAM) is a receptor for a lymphotropic strain of measles virus and may account for the immunosuppressive properties of this virus. Virology.

[13] Sato H., Yoneda M., Honda T., Kai C. (2012). Morbillivirus receptors and tropism: multiple pathways for infection. Front Microbiol.

[14] Pratakpiriya W., Seki F., Otsuki N., Sakai K., Fukuhara H., Katamoto H. (2012). Nectin4 is an epithelial cell receptor for canine distemper virus and involved in neurovirulence. J Virol.

[15] Noyce R.S., Delpeut S., Richardson C.D. (2013). Dog nectin-4 is an epithelial cell receptor for canine distemper virus that facilitates virus entry and syncytia formation. Virology.

[16] Noyce R.S., Richardson C.D. (2012). Nectin 4 is the epithelial cell receptor for measles virus. Trends Microbiol.

[17] Noyce R.S., Bondre D.G., Ha M.N., Lin L.T., Sisson G., Tsao M.S. (2011). Tumor cell marker PVRL4 (nectin 4) is an epithelial cell receptor for measles virus. PLoS Pathog.

[18] Tahara M., Ohno S., Sakai K., Ito Y., Fukuhara H., Komase K. (2013). The receptor-binding site of the measles virus hemagglutinin protein itself constitutes a conserved neutralizing epitope. J Virol.

[19] Rima B.K., Wishaupt R.G., Welsh M.J., Earle J.A. (1995). The evolution of morbilliviruses: a comparison of nucleocapsid gene sequences including a porpoise morbillivirus. Vet Microbiol.

[20] Barrett T. (1999). Morbillivirus infections, with special emphasis on morbilliviruses of carnivores. Vet Microbiol.

[21] de Swart R.L., Yuksel S., Langerijs C.N., Muller C.P., Osterhaus A.D. (2009). Depletion of measles virus glycoprotein-specific antibodies from human sera reveals genotype-specific neutralizing antibodies. J Gen Virol.

[22] Seki F., Ono N., Yamaguchi R., Yanagi Y. (2003). Efficient isolation of wild strains of canine distemper virus in Vero cells expressing canine SLAM (CD150) and their adaptability to marmoset B95a cells. J Virol.

[23] Adombi C.M., Lelenta M., Lamien C.E., Shamaki D., Koffi Y.M., Traore A. (2011). Monkey CV1 cell line expressing the sheep-goat SLAM protein: a highly sensitive cell line for the isolation of peste des petits ruminants virus from pathological specimens. J Virol Methods.

[24] de Vries R.D., Ludlow M., Verburgh R.J., van Amerongen G., Yuksel S., Nguyen D.T. (2014). Measles vaccination of non-human primates provides partial protection against infection with canine distemper virus. J Virol.

[25] de Swart R.L., Yuksel S., Osterhaus A.D. (2005). Relative contributions of measles virus hemagglutinin- and fusion protein-specific serum antibodies to virus neutralization. J Virol.

[26] Frecha C., Costa C., Negre D., Gauthier E., Russell S.J., Cosset F.L. (2008). Stable transduction of quiescent T cells without induction of cycle progression by a novel lentiviral vector pseudotyped with measles virus glycoproteins. Blood.

[27] Whitt M.A. (2010). Generation of VSV pseudotypes using recombinant DeltaG-VSV for studies on virus entry, identification of entry inhibitors, and immune responses to vaccines. J Virol Methods.

[28] Terio K.A., Craft M.E. (2013). Canine distemper virus (CDV) in another big cat: should CDV be renamed carnivore distemper virus. mBio.

[29] Williams E.S., Thorne E.T., Appel M.J., Belitsky D.W. (1988). Canine distemper in black-footed ferrets (*Mustela nigripes*) from Wyoming. J Wildl Dis.

[30] Roelke-Parker M.E., Munson L., Packer C., Kock R., Cleaveland S., Carpenter M. (1996). A canine distemper virus epidemic in Serengeti lions (*Panthera leo*). Nature.

[31] Origgi F.C., Sattler U., Pilo P., Waldvogel A.S. (2013). Fatal combined infection with canine distemper virus and orthopoxvirus in a group of Asian marmots (*Marmota caudata*). Vet Pathol.

[32] Mamaev L.V., Visser I.K., Belikov S.I., Denikina N.N., Harder T., Goatley L. (1996). Canine distemper virus in Lake Baikal seals (*Phoca sibirica*). Vet Rec.

[33] Alexander K.A., Kat P.W., Frank L.G., Holekamp K.E., Smale L., House C. (1995). Evidence of canine distemper virus infection among free-ranging spotted hyenas (*Crocuta crocuta*) in the Masai Mara, Kenya. J Zoo Wildlife Med.

[34] Seimon T.A., Miquelle D.G., Chang T.Y., Newton A.L., Korotkova I., Ivanchuk G. (2013). Canine distemper virus: an emerging disease in wild endangered Amur tigers (*Panthera tigris altaica*). mBio.

[35] Hvistendahl M. (2015). Endangered species. Captive pandas succumb to killer virus. Endangered species. Captive pandas succumb to killer virus. Science.

[36] Munir M., Zohari S., Berg M. (2013). Epidemiology and distribution of peste des petits ruminants. molecular biology and pathogenesis of peste des petits ruminants virus.

[37] Banyard A.C., Wang Z., Parida S. (2014). Peste des petits ruminants virus, eastern Asia. Emerg Infect Dis.

[38] Lloyd-Smith J.O. (2013). Vacated niches, competitive release and the community ecology of pathogen eradication. Philos Trans R Soc Lond B: Biol Sci.

[39] Banyard A.C., Parida S., Batten C., Oura C., Kwiatek O., Libeau G. (2010). Global distribution of peste des petits ruminants virus and prospects for improved diagnosis and control. J Gen Virol.

[40] Graham F.L., Smiley J., Russell W.C., Nairn R. (1977). Characteristics of a human cell line transformed by DNA from human adenovirus type 5. J Gen Virol.

[41] Yasumura Y., Kawakita Y. (1963). Studies on the SV40 virus in tissue cultures. Nippon Rinsho.

[42] Carpenter M.A., Appel M.J.G., Roelke-Parker M.E., Munson L., Hofer H., East M. (1998). Genetic characterization of canine distemper virus in Serengeti carnivores. Vet Immunol Immunopathol.

[43] Harder T.C., Kenter M., Vos H., Siebelink K., Huisman W., van Amerongen G. (1996). Canine distemper virus from diseased large felids: biological properties and phylogenetic relationships. J Gen Virol.

[44] Gillespie J.H., Rickard C.G. (1956). Encephalitis in dogs produced by distemper virus. Am J Vet Res.

[45] von Messling V., Springfeld C., Devaux P., Cattaneo R. (2003). A ferret model of canine distemper virus virulence and immunosuppression. J Virol.

[46] Takada A., Robison C., Goto H., Sanchez A., Murti K.G., Whitt M.A. (1997). A system for functional analysis of Ebola virus glycoprotein. Proc Natl Acad Sci U S A.

[47] Naldini L., Blomer U., Gallay P., Ory D., Mulligan R., Gage F.H. (1996). In vivo gene delivery and stable transduction of nondividing cells by a lentiviral vector. Science.

[48] Hu Q., Chen W., Huang K., Baron M.D., Bu Z. (2012). Rescue of recombinant peste des petits ruminants virus: creation of a GFP-expressing virus and application in rapid virus neutralization test. Vet Res.

[49] Lorenz R.J., Bogel K. (1973). Laboratory techniques in rabies: methods of calculation. Monogr Ser World Health Org.

[50] Appel M., Robson D.S. (1973). A microneutralization test for canine distemper virus. Am J Vet Res.

[51] Bussell R.H., Karzon D.T. (1965). Canine distemper virus in ferret, dog and bovine kidney cell cultures. Arch Gesamte Virusforsch.

[52] Mather S.T., Wright E., Scott S.D., Temperton N.J. (2014). Lyophilisation of influenza, rabies and Marburg lentiviral pseudotype viruses for the development and distribution of a neutralisation-assay-based diagnostic kit. J Virol Methods.

[53] Tahara M., Ito Y., Brindley M.A., Ma X., He J., Xu S. (2013). Functional and structural characterization of neutralizing epitopes of measles virus hemagglutinin protein. J Virol.

[54] Zhang X., Wallace O.L., Domi A., Wright K.J., Driscoll J., Anzala O. (2015). Canine distemper virus neutralization activity is low in human serum and it is sensitive to an amino acid substitution in the hemagglutinin protein. Virology.

[55] Goldstein T., Mazet J.A.K., Gill V.A., Doroff A.M., Burek K.A., Hammond J.A. (2009). Phocine distemper virus in Northern Sea Otters in the Pacific Ocean, Alaska, USA. Emerg Infect Dis.

[56] van de Bildt M.W., Martina B.E., Vedder E.J., Androukaki E., Kotomatas S., Komnenou A. (2000). Identification of morbilliviruses of probable cetacean origin in carcases of Mediterranean monk seals (*Monachus monachus*). Vet Rec.

[57] de Swart R.L., Duprex W.P., Osterhaus A.D. (2012). Rinderpest eradication: lessons for measles eradication. Curr Opin Virol.

[58] Bieringer M., Han J.W., Kendl S., Khosravi M., Plattet P., Schneider-Schaulies J. (2013). Experimental adaptation of wild-type canine distemper virus (CDV) to the human entry receptor CD150. PLoS ONE.

