# Supplementary material for: Efficient generation of vesicular stomatitis virus (VSV)-pseudotypes bearing morbilliviral glycoproteins and their use in quantifying virus neutralising antibodies
Source: Vaccine. 2016 Feb 3;34(6):814–22. doi: 10.1016/j.vaccine.2015.12.006 (PMC4742518; doi:10.1016/j.vaccine.2015.12.006)
Supplement: Supplementary file 1 [file mmc1.pptx]

## Slide 1
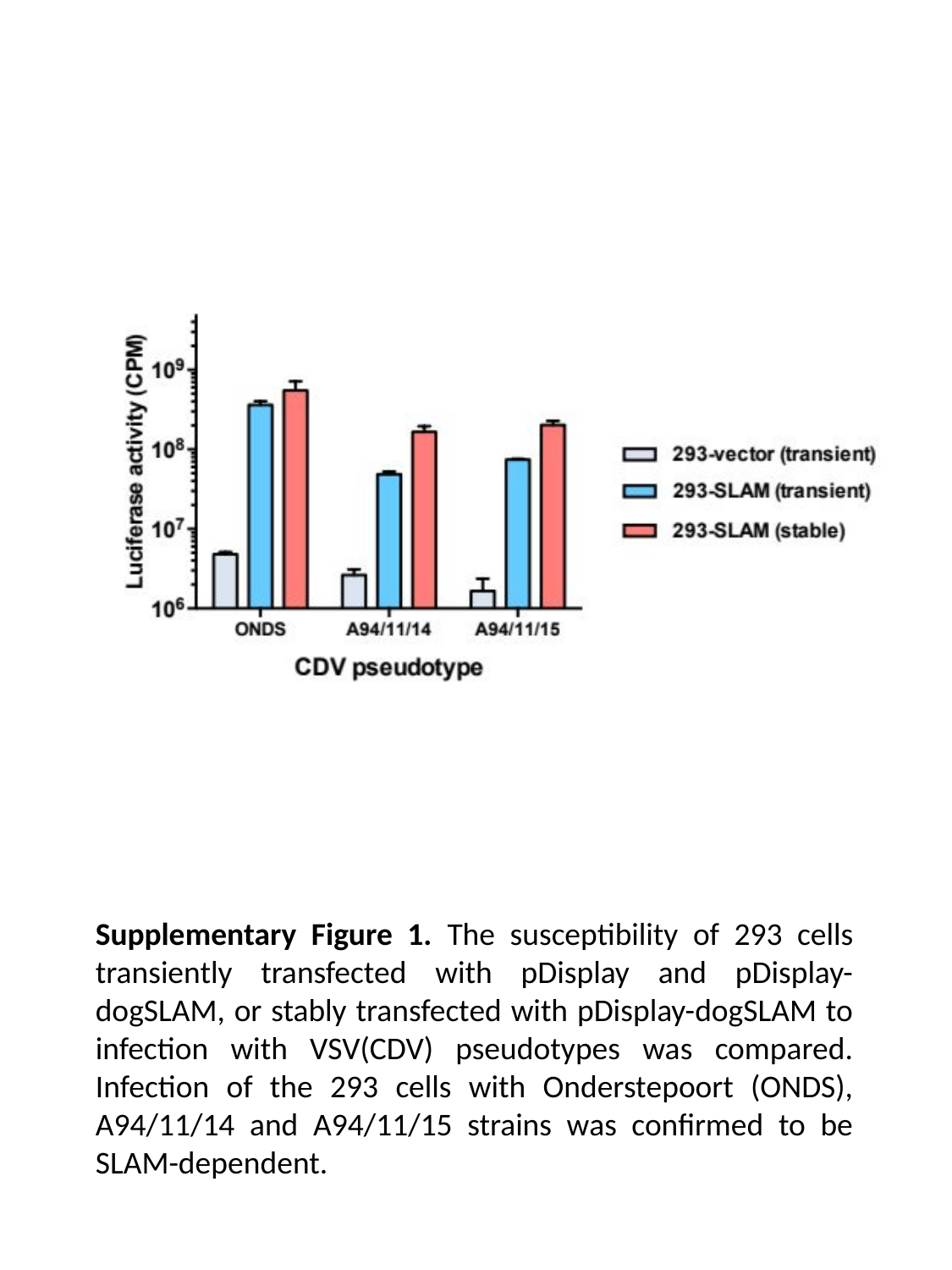

Supplementary Figure 1. The susceptibility of 293 cells transiently transfected with pDisplay and pDisplay-dogSLAM, or stably transfected with pDisplay-dogSLAM to infection with VSV(CDV) pseudotypes was compared. Infection of the 293 cells with Onderstepoort (ONDS), A94/11/14 and A94/11/15 strains was confirmed to be SLAM-dependent.
